# Palladium Nanoparticles: Toxicological Effects and Potential Implications for Occupational Risk Assessment

**DOI:** 10.3390/ijms19020503

**Published:** 2018-02-07

**Authors:** Veruscka Leso, Ivo Iavicoli

**Affiliations:** Section of Occupational Medicine, Department of Public Health, University of Naples Federico II, Via S. Pansini 5, 80131 Naples, Italy; veruscka.leso@gmail.com

**Keywords:** palladium nanoparticles, ecotoxicity, cytotoxic effects, in vivo toxicity, biomarkers of exposure, biomarkers of effect, risk assessment, risk management

## Abstract

The increasing technological applications of palladium nanoparticles (Pd-NPs) and their consequent enhancing release into the community and occupational environments, have raised public health concerns regarding possible adverse effects for exposed subjects, and particularly for workers chronically and highly exposed to these materials, whose toxico-kinetic and dynamic behavior remains to be fully understood. Therefore, this review aimed to critically analyze literature data to achieve a more comprehensive knowledge on the toxicological profile of Pd-NPs. Results from available studies demonstrated the potential for these chemicals to affect the ecosystem function, to exert cytotoxic and pro-inflammatory effects in vitro as well as to induce early alterations in different target organs in in vivo models. However, our revision pointed out the need for future studies aimed to clarify the role of the NP physico-chemical properties in determining their toxicological behavior, as well as the importance to carry out investigations focused on environmental and biological monitoring to verify and validate experimental biomarkers of exposure and early effect in real exposure contexts. Overall, this may be helpful to support the definition of suitable strategies for the assessment, communication and management of Pd-NP occupational risks to protect the health and safety of workers.

## 1. Introduction

Modern science has experienced one of its major breakthroughs with nanotechnology in the last decades. This enabled nano-dimensional materials (in the 1–100 nm size domain) to be produced and applied in a number of technological and consumer fields due to their peculiar physico-chemical properties [1].

Metallic based nanomaterials have attracted great scientific attention in the nanotechnology field. Particularly, given that noble metals are characterized by remarkable catalytic, electronic, magnetic, optical, and mechanical properties per se [2,3,4], the study of the characteristics and possible uses of nano-sized noble metal materials has emerged as hugely important and valuable for the benefits potentially offered in a number of different applications.

In this regard, palladium (Pd) is a rare and precious metal that belongs to the Platinum group elements. It is largely employed as an active catalyst material in automotive catalytic converters, but finds also application in the electronic, engineering, biomedical, and jewelry sectors [5,6,7,8]. Palladium-NPs offer the opportunity for being even more effective catalyst materials due to their high surface area to volume ratio and high surface energy [9].

Therefore, the emerging applications of these NPs, as detailed in the next paragraph, and the consequent enhanced likelihood of human exposure due to the release of such materials in living and occupational environments, raise public health and scientific concerns over possible environmental and human health implications [10]. From an occupational health perspective, this seems an even more urgent issue to assess, since workers may become highly and chronically exposed to these still not-fully explored nanomaterials. In this context, it is important to achieve a deeper and more comprehensive understanding of the inherent toxicity of Pd-NPs, with the purpose of a safe handling and use of these innovative materials. Therefore, the aim of this review was to revise potential applications of Pd-NPs and critically assess available knowledge concerning possible toxicological effects induced by Pd-NPs on in vitro, ex vivo and in vivo models in order to extrapolate critical issues important for understanding potential risks for workers.

## 2. Possible Pd-NP Applications

Palladium-NPs are characterized by invaluable catalytic, mechanical and optical properties which may offer the opportunity for their employment in a number of industrial applications. In this regard, Pd-NPs have been extensively investigated for a wide range of chemical applications [11]. Their catalyst potential, related to the ability to dissociate molecular hydrogen into the atomic state, has been applied in organic carbon–carbon coupling formation, i.e., the Suzuki–Miyaura and the Heck cross-coupling reactions [12,13,14,15], hydrogenations of unsaturated olefins [13], and alcohol oxidations [16]. Overall, this potential has been successfully employed to control the hazardous exhaust emissions from automotive engines, to process environmental pollutants, as well as to produce intermediates of pharmaceuticals and fine chemicals [17,18]. Additionally, Pd-NPs exhibited excellent degradation of methylene blue, methyl orange and nitrophenol dyes, and therefore, they can be employed to treat the effluents polluted with such compounds [11].

Apart from the organic catalysis, Pd nanomaterials have been widely used in electro-catalysis. The property of Pd to adsorb hydrogen has led Pd-NPs to be employed in hydrogen storage [19,20], and electrochemical reactions in fuel cells [21]. Carbon microspheres-supported Pd-NPs possess excellent electro-catalytic properties and may be of great potential in direct ethanol fuel cells [22]. palladium nanostructures have gained interest in the last decade also in a number of other applications including the composition of electrical equipment [23,24] or as sensors for the detection of various analytes [20], i.e., Pd-NPs doped with chitosan–graphene have been employed as biosensors for glucose estimation [25]. In medicine, Pd nanosheets and Pd-NPs have been investigated for photothermal therapy and as anti-tumor/anti-microbial agents, although they have still not been practically exploited in this therapeutic fields [17].

## 3. In Vitro Studies

A number of studies reported the ability of Pd-NPs to exert eco-toxicological adverse effects on seed germination [26], pollen [27] and plant growth [28]. Significant cytotoxic effects were also detected in several human cellular models, including respiratory cells [29,30], peripheral blood [31], and cervical [32,33,34], liver [35], ovarian [4] as well as skin melanoma cancer cells [36], although no unique modes of action could be defined. Additionally, a pro-inflammatory action of Pd-NPs was demonstrated by increased levels of cytokines as key mediators of such effect [29,37,38] (Table 1).

### 3.1. Plant Models

The emerging application of Pd-NPs in many fields inevitably led these chemicals to contaminate environmental matrices, potentially resulting in bioaccumulation in living organisms. This raised interest in assessing the effects of such metal-NPs on plant models. In this regard, Battke et al. [28] provided the first report regarding the plant uptake of Pd-NPs and their phyto-toxic effects. As a sign of abiotic stress, leaf length of barley plants decreased or became extremely variable, and leaves developed as rigid and slightly convoluted in response to Pd-NP treatment.

Palladium-NPs were also reported to significantly affect the growth of lettuce seeds, as measured by shoot to root ratios, when these were planted in soil samples previously incubated with NPs for 15 days [26]. Conversely, Pd-NPs supported on halloysite nanotubes showed no influence on radish germination and development of high and low vigor seeds of *Raphanus sativus*, mitotic index and chromosomal figures, therefore supporting the biological safety of such nanomaterials [39]. Speranza et al. [27] assessed the effects of Pd-NPs on the performance of kiwifruit pollen cultures, as an experimental model largely employed to assess the chemical impact on plant cell metabolism. Palladium-NPs were able to induce morphological alterations of pollen grains, damage to cell membranes, and depletion of endogenous calcium, therefore resulting in impaired pollen tube emergence and growth. When NP induced effects were compared to those exerted by PdCl_2_ on the same endpoints, higher cyto-toxicity was reported for the nano-sized form, maybe in relation to a greater metal adsorption derived from NP exposure.

### 3.2. Animal Cells

Cell viability tests with RTgill-W1 (*Oncorhynchus mykiss*) fish cell lines showed no obvious acute toxicity for Pd/magnetite NPs. Metabolic activity and membrane integrity, as cell viability parameters, showed a slight decreasing trend after 1 h of particle exposure. However, this was attributed to a cellular stress response induced by NP addition, more than to a specific cytotoxic effect of NPs. After three days of exposure, cell viability was fully recovered. The third endpoint under study, lysosomal integrity, did not show any alteration at both periods investigated [40].

Conversely, in the normal diploid Rat-1 fibroblast cell line acutely and sub-acutely treated with Pd-NPs, a significant dose and time-dependent cell growth inhibition was observed [30]. A cell cycle arrest with accumulation of cells in G0/G1 phases and a significant DNA damage was evident, although only a slight increase in the intracellular reactive oxygen species (ROS) levels could be detected.

### 3.3. Human Cells

#### 3.3.1. Cytotoxic Effects

The exposure to Pd-NPs was reported to induce different toxic effects on various cellular models. The assessment of cell viability in human colon adenocarcinoma Caco-2 cells and HaCaT keratinocytes, treated with Pd-NPs, revealed only minor effects, maybe related to the cellular stress caused by the NP application [40]. On the other hand, Wilkinson et al. [29] showed that the exposure of primary bronchial epithelial cells (PBECs), and lung carcinoma epithelial cells (A549) used to simulate the upper and lower respiratory tract, respectively, to Pd-NPs resulted in a concentration-dependent cytotoxicity. Of note, PBECs were markedly more affected by Pd-NPs than A549 cells. In line with these findings, apoptosis was induced in a dose-dependent manner by Pd-NPs in PBECs, but not in A549 cells [29].

Nanoparticle chemical composition appeared also relevant to determine cellular viability effects, since individual Pd-NPs resulted in less cytotoxicity compared to bimetallic Pt-Pd-NPs on human epithelial cervical cancer HeLa cells [32]. Such enhanced effect could be attributed to a synergistic action of both components. Also, the type of NP surface coating was reported as a possible influencing factor for the Pd-NP toxicological profile. Positively charged cysteamine functionalized Pd nanosheets, in fact, showed a much higher cytotoxicity on human liver HepG2 cancer cells, compared to negatively charged 3-mercaptopropionic acid functionalized NPs [35]. This was supposed to be related to the stronger contact that two dimensional, cysteamine functionalized nanosheets may have with cell surface compared to the mercaptopropionic-acid nanosheets, which aggregated in three dimensional particles, therefore changing their contact level with the cellular membrane. However, a dose-dependent cytotoxic effect on HepG2 cells was also reported by Rajakumar et al. [33] with Pd-NPs synthesized using the aqueous leaf extracts of *Eclipsa prostrata*. Increasing concentrations were able to induce cytopathic effects leading to cell damage and necrosis.

When Pd-NP acute and sub-acute cytotoxic effects were investigated on peripheral blood mononuclear cells (PBMCs) leaving quiescence through a phytohemagglutinin-L stimulation, a time- and dose-dependent reduction in cell viability could also be detected [31]. Interestingly, non-cytotoxic Pd-NP doses caused a significant increase of cells within the G0/G1-phase, as demonstrated by an increased percentage of mitogen activated PBMCs with a diploid DNA content, and a significant reduction of cells in GS- and G2/M-phases. In line with these results [31], a time and dose-dependent inhibition of cell growth has been recently demonstrated by Iavicoli et al. [30] in exponentially growing cultures of A549 cells treated with Pd-NPs for sub-acute periods of time (48–120 h). However, such cell viability inhibition was less evident compared to that determined in the same study on Rat-1 fibroblasts, suggesting a species-specific susceptibility to the NP toxicity. Moreover, Pd-NP-induced inhibition of cell growth was not associated with the induction of apoptosis. As reported by Petrarca et al. [31], Pd-NPs induced a progressive cell cycle arrest with accumulation of cells in the G0/G1 phase of the cell cycle, which could suggest a potential toxic effect of Pd-NPs on DNA. According to this statement, the authors found that Pd-NPs were able to induce a significant increase in DNA breaks already after 4 h of exposure and such damages become even more evident after longer periods of treatment even if only a slight increase in the intracellular ROS levels could be detected. Alarifi et al. [36] demonstrated that Pd-NPs not only inhibited human skin malignant melanoma A375 cell proliferation in a dose- and time-dependent manner, but also induced apoptosis, DNA damage and a cell cycle arrest. Inhibition of cell growth was specifically related with a reduction of the percentage of cells in the G0/G1 phase and an accumulation of those in the S and G2/M phases of the cell cycle.

A recent study investigated the possible effects that Pd-NPs exerted on human eosinophil cells known to have a key role in lung diseases and allergies [43]. In contrast to previous findings, Pd-NPs did not induce necrotic or apoptotic cellular viability alterations in both human 3D10 eosinophils and primary eosinophil cells in vitro. However, these NPs were able to induce actin cytoskeleton rearrangement in exposed cells, therefore determining their increased adhesion to endothelial cells. This was confirmed by the pre-treatment of cells with cytochalasin D, a potent inhibitor of actin polymerization, which in turn determined levels of adhesion comparable to those found in untreated controls.

Concerning possible modes of action, oxidative stress is considered as one of the most important mechanisms for cytotoxic effects of NPs [44]. However, regarding Pd-NPs, an oxidative stress induction was detected only in A375 human skin malignant melanoma cells [36] and in A2780 human ovarian cancer cells [4] based on ROS production following an acute or sub-acute treatment, which could be responsible for the induction of DNA damage and apoptotic-mediated cell death. Gurunathan et al. [4] also hypothesized that excessive production of ROS could trigger an autophagic phenomenon in ovarian cancer cells, eventually leading to cytotoxicity. Interestingly, the surface-specific ROS production of Pd-NPs was reported as being significantly related to the primary particle size, with a maximum around 12 nm, for both acellular and human leukaemia cellular environments [41]. On the other side, no significant ROS production was observed for Pd-NPs in Caco-2 and Hacat [40], A549 cells [29,30], PBECs [29], PBMCs [31], and human eosinophils [43].

Therefore, other possible molecular mechanisms should be elucidated for their role in Pd-NP cytotoxicity. Dahal et al. [34] confirming these results, demonstrated that Pd-nanocubes induced apoptosis in transformed human epithelial HeLa cells only in serum-free medium. The effect of serum on compromising the efficacy of Pd-nanocubes in inducing apoptosis can be related to the healing properties of proteins, antibodies and antigens present in serum on cytotoxic effects, as well as on the possibility that these components may form different bio-molecular coronas around nanocubes changing their biological reactivity. Apoptosis was the primary route of Pd-nanocube-induced cell death, although it was associated with a hyperpolarization of mitochondria, contrary to common depolarization initiated by ROS. This may suggest that the mechanism of Pd-nanocube toxicity was different from cytotoxic effects mediated by oxidative stress reactions.

Regarding the role of solubility in NP-induced effects, Petrarca et al. [31] suggested that Pd ions, per se or released from NPs could be the inducers of Pd toxicity as assessed comparing effects induced by Pd(IV) ions and Pd-NPs on PBMCs. Pd ions resulted more toxic, compared to Pd-NPs, causing almost complete loss of cell viability earlier, and at a lower dose, and a significant ROS increase. Following Pd (IV) ion exposure, more marked subcellular alterations, i.e., the presence of numerous auto-phagosomal vacuoles containing damaged mitochondria, and/or undigested cytoplasmic material, and a significant amplification of cell cycle alterations described for Pd-NPs, could be demonstrated. This may suggest that Pd ions released from Pd-NPs may have a role in their toxicological behavior. Metal ions may accumulate in mitochondria damaging their function which may in turn arrest the cell cycle and cause cell death. However, deeper research is necessary to verify and confirm such possible mechanism of action.

#### 3.3.2. Immunological and Inflammatory Responses

The immune potential of Pd-NPs was evaluated in a couple of studies through the analysis of cytokine release and expression in PBMCs of non-atopic [37] and Pd-sensitized women [38]. In the first study [37], a significant reduction in IL-17 and TNF-α concentrations was induced by NP exposure, with or without lipopolysaccharide (LPS) stimulation, while the INF-γ release was significantly enhanced by the highest concentration of NPs in LPS stimulated cells. These results were partially comparable to those obtained with a Pd salt in the same conditions of exposure, with the exception of the Pd-NP stimulatory action on the INF-γ release. Overall, this may suggest that Pd-NPs may exert an important immune modulatory effect in vitro which was also confirmed when such cytokine changes were investigated in PBMCs obtained from Pd-sensitized non-atopic volunteers [38]. The addition of 10^−5^ M Pd-NPs did not significantly modify the release of cytokines in LPS un-stimulated cultures, but induced a significant inhibition of TNF-α release in LPS stimulated PBMCs. These findings suggested a possible immuno-modulatory effect of Pd-NPs on cytokine release. However, deeper investigation is necessary to define underlying modes of action in PBMCs from Pd-sensitized or not-sensitized subjects and possible long-term health implications of such alterations.

Interleukin-8 secretion was also investigated by Wilkinson et al. [29] as a possible biomarker of the inflammogenic potential of Pd-NPs on PBECs and A459 cells. Additionally, the levels of prostaglandin E_2_ (PGE_2_), potentially involved in a number of biological functions, including vasodilatation and both anti- and pro-inflammatory action, were also evaluated. A non-linear trend could be detected for the secretion of IL-8 in response to Pd-NP treatment by A549 lung epithelial cells. A dose-dependent decrease was evident in the lower concentration range (0.01–1 µg/mL), while a significant increase compared to controls was achieved at the highest concentration (10 µg/mL). On the other hand, a dose-dependent decrease could be detected for the PGE_2_ secretion in both cell types. Notably, these effects are seen at a non-cytotoxic concentration of Pd-NPs, indicating that these biomarkers serve as sensitive indicators of the biological effects of Pd-NPs. Recently, a significant, dose-dependent increase in IL-8 secretion was also reported in a three-dimensional cellular model obtained co-culturing PBECs and human fetal lung fibroblasts MRC-5 at an air liquid interface, able to mimic an in vivo healthy and chronic bronchitis-like mucosa acutely exposed to Pd-NPs [42]. This demonstrated that Pd-NPs could induce an inflammatory response in both mucosal models, although IL-8 concentrations were significantly higher in the inflamed model compared to the normal one.

## 4. Ex Vivo Studies

Skin contact with Pd can result in sensitization and allergic contact dermatitis [45,46,47]. The Pd-NP higher surface area to mass ratio may be responsible for greater biological activities compared to the bulk form of the metal, as well as an easier release of reactive metal ions [48,49], with a greater potential for skin permeation. To verify this latter aspect, Larese et al. [50] investigated the penetration of 0.60 mg/cm^2^ of 10.7 ± 2.8 nm sized Pd-NPs applied on intact and needle-damaged human skin pieces obtained as a surgical waste from four different donors in an in vitro diffusion cell system. Human skin absorption of Pd-NPs occurred in both intact and damaged skin, although lesions increased the metal absorption significantly. The Pd content in intact skin decreased significantly from the epidermis to the dermis. Inside the skin, these NPs can be a long-term source of metal that could be involved in the sensitization process or can be available for systemic diffusion.

## 5. In Vivo Studies

Several biological models, from bacterial strains [51] to mammalian organisms were reported to be affected by Pd-NP treatment. The immune [52,53], renal [54] and endocrine systems [55] were demonstrated as critical targets for the toxic action of Pd-NPs (Table 2). The elemental Pd content in urine, and changes in cytokine serum levels, urinary protein content and hormonal serum concentrations, emerged as possible biomarkers of Pd-NP exposure and early effect, although further studies are necessary to confirm these preliminary results and validate their possible employment in real exposure settings.

### 5.1. Bacteria and Microbial Communities

To understand the interactions between nanomaterials and micro-organisms can be helpful to define NP toxicological profiles on simple biological entities and extrapolate information to be verified on more advanced organisms within more complex environmental conditions of exposure. In this regard, an anti-microbial effect of Pd-NPs was reported by Adams et al. [51] toward Gram negative *Escherichia coli* and Gram-positive *Staphylococcus aureus* bacterial cultures, although with a greater inhibition in this latter strain, maybe in relation to the stronger resistance of the cell wall of negative bacteria compared to positive ones. Interestingly, the anti-bacterial activity resulted dependent on the NP size, and time-periods investigated. Antibacterial properties of Pd-NPs were also subsequently confirmed on *E. coli* and *S. aureus* cultures [58] and on the novel multidrug resistant *Cronobacter sakazakii* strain in which NPs were able to eradicate also biofilm bacterial growth, responsible for their multidrug resistance [60]. A comparable antimicrobial impact was also evident with bimetallic-Pd-iron-NPs, which demonstrated to significantly inhibit the growth rate of the dyphenyl ether degrading bacterial strain *Sphingomonas* sp. PH-07 in a dose-dependent manner, at concentrations greater than 0.1 g/L [56].

In order to address whether the known antibacterial properties of the conducting polymer polyaniline [61], could be enhanced via the incorporation with metal NPs, Pt-Pd colloidal solutions as well as Pt-Pd-polyaniline nanocomposites were explored for their action against selective Gram positive (*Streptococcus* sp. and *Staphylococcus* sp.) and Gram-negative bacteria (*E. coli* and *Klebsiella* sp.) [57]. Nanocomposite materials exhibited improved antibacterial activity compared to pristine polyaniline. Interestingly, Pt-Pd individual nano-metal colloids did not exhibit any antibacterial activity, suggesting that the enhanced biological impact could be dependent on the interaction between metal NPs and a pristine polyaniline matrix as well as on the greater reactivity of smaller NPs in the nano-composites. The possibility to employ Pd-NPs in the remediation of groundwater, wastewater and sediments, raised concerns among possible effects these NPs may exert on environmental microbial communities once released into the environment [59]. Therefore, the impact of Pd-NPs on the marine bacteria was assessed on a single eco-toxicological model of marine bacterium, *V. fischeri*, as well as on active bio-remediating microbial communities incubated under laboratory biogeochemical conditions mimicking those occurring in situ. Pd-NPs showed nor toxic effects on *V. fischeri*, nor alterations in the total bacterial community structure, as its biodiversity was increased compared to the not exposed community. Additionally, concerning respiratory metabolisms, the negligible effect on organohalide respiration activities, as well as the dose-dependent inhibition of sulfate reduction and methanogenesis, further support the lack of ecotoxic effects of Pd-NPs on marine microbiome. Comparably, no significant effects on the number of colony forming units or on the total soil community metabolic fingerprint could be detected in soil microbial communities exposed to Pd-NPs in experimental microcosm conditions of exposure serving as a direct analog of ecological system [26]. No differences could be also detected between the variable NP concentrations employed, maybe in relation to the immobilization of NPs on soil organic compounds, such as humic acids.

Additionally, the antiplasmodial activity of Pd-NPs was demonstrated by Rajakumar et al. [33] in mice intraperitoneally inoculated with *Plasmodium berghei*-infected red blood cells. The untreated controls showed a progressively increasing parasitemia, while animals treated via the oral route with Pd-NPs showed a 78.13% decrease in parasitemia with a reported inhibitory concentration 50 value of 16.44 mg/kg/body weight.

### 5.2. Animal Models 

Concerning in vivo results, investigations conducted on female Wistar rats, proved the ability of Pd-NPs to significantly affect the immune system of treated animals inducing alterations in the serum levels of several cytokines secreted by different T helper (Th) lymphocyte subsets [52,53]. A subacute (14 days) exposure to increasing doses of Pd-NPs was able to induce a general, although not significant, upward trend, in all cytokine serum concentrations investigated, while a significant increase was only determined by the highest dose of Pd-NPs (12 μg/kg) [52]. These findings may suggest that Pd-NPs could exert a significant and generalized stimulatory impact on the immune system of exposed rats. However, when investigated in a sub-chronic time frame, such cytokine increasing trend was not more evident [53]. Repeated injections of Pd-NPs, in fact, induced a decreasing trend in serum levels in most of the cytokines investigated, with the highest concentration (12 µg/kg) determining significant inhibitory effects compared to controls, supporting a more complex interaction between these nanomaterials and the immune system, related also to the time points investigated.

As another possible target organ of the Pd-NP toxic action, the effects on the renal system was recently assessed in Wistar rats acutely exposed via the intravenous route to a wide range of NP concentrations. Results showed an increasing trend in urinary Pd levels demonstrating that Pd urinary concentrations were directly associated with the administered doses. Additionally, urinary concentration of low, retinol binding protein and β2-microglobulin, and high molecular weight proteins were employed to assess the nephrotoxic action of Pd-NPs [54]. Interestingly, the highest administered dose (12 µg/kg) was able to induce a significant increase in urinary retinol binding protein and β2-microglobulin levels compared to controls. This may suggest that Pd-NPs may exert a nephrotoxic action at the renal tubular level. In fact, these low molecular weight proteins are excreted in urine in small quantities when tubular function is normal, while an increase in their urinary excretion is indicative of renal tubular damage. As a confirmation of this specific damage, ultra-structural analysis of kidney specimens revealed subcellular dose-dependent morphological alterations with the main target in the proximal and distal tubular epithelium. However, these preliminary results need to be confirmed also to define possible Pd-NP molecular mechanisms of action.

More recently, a first attempt to investigate the endocrine disruptive potential of Pd-NPs was performed by our group of research in an animal model of sexually mature female Wistar rats sub-acutely exposed to NPs via a single tail vein injection [55]. An abnormal function of the hypothalamic–pituitary–gonadal axis was detected as demonstrated by the reduced serum concentrations of estradiol (E2) and testosterone (T) and the increased level of luteinizing hormone (LH) in treated animals compared to controls, which become significant at the highest exposure dose (12 µg/kg). Overall, these results demonstrated the ability of Pd-NPs to significantly affect the normal sex hormone levels, thus suggesting that they may play an important role in disrupting the physiological functions of the female reproductive system of Wistar rats.

## 6. Discussion

Over these last years, the enhancing interest in the synthesis and application of Pd-NPs, together with their consequent increasing release into the community and occupational environments, have raised concerns regarding possible adverse effects exerted on ecosystems and human health. Particular attention has been focused on the health of workers who may be repeatedly and long-term exposed to high doses of these nanomaterials, whose toxico-kinetic and dynamic behavior is still not fully understood. In this emerging scenario, this is the first attempt to comprehensively analyze the state of the art concerning Pd-NP toxicological behavior on simple and more complex biological systems in order to extrapolate information useful to guide suitable risk assessment processes and point out future research needs.

Although no definite conclusions can be drawn concerning the eco-toxicological impact of Pd-NPs, the adverse effects reported on plant/seed models in vitro [27,28,39] do not allow to exclude a potentially serious risk for plant reproductive success and growth, as well as for the ecosystem functions.

Regarding Pd-NP antimicrobial properties, a significant inhibitory effect on bacterial growth was evident in pure microbial cultures [51,60] while it was not evident in more complex soil and marine microbial communities under experimental conditions mimicking those of the natural environment [26,59]. This may be dependent on the possible interactions that NPs may have with environmental compounds that may, in turn, prevent strong influences on microbial communities. Overall, these data point out the importance of combining standard eco-toxicity tests, microbial metabolic assessments, and analyses of microbial community composition, in a multifaced approach to evaluate the impact of NPs on the environmental microbiota and underlined molecular mechanisms of action [59].

The majority of the reviewed in vitro studies reported significant cytotoxic [4,29,30,31,34,36] and immuno-modulatory effects [29,37,38] induced by Pd-NP exposure on diverse animal and human cells. Interestingly, different responses to NP treatment were determined in cell cultures used as models for the upper and lower part of the respiratory tract, maybe in relation to the differences in particle uptake, and culture media employed, which may change the NP bio-molecular corona, therefore affecting NP toxicological profiles [29]. In this scenario, more complex tri-dimensional in vitro models, resembling healthy and chronic bronchitis-like mucosa, may be useful to achieve data to be easily extrapolated to an in vivo context and useful to define areas of the respiratory tract, as well as pathological conditions, characterized by a greater susceptibility to the NP toxicity [42].

Interestingly, in vitro studies are generally useful to define possible NP molecular mechanisms of action. However, current evidence on Pd-NPs do not allow to define specific conclusions in this regard, as no uniform results were reported concerning the role of such chemicals as triggers of oxidative stress reactions, apoptotic pathways, DNA damage cascades as well as cell cycle disturbances. Additionally, it cannot be excluded that the type, i.e., organic ligands, surfactants, polymers and dendrimers, and variable concentrations of chemical stabilizers employed in NP preparation, may influence their toxicological profile. Therefore, further investigation seems necessary to define the role that the complex interplay between the different NP physico-chemical properties, cellular growth media and peculiar cellular-type features may have in determining different patterns of cytotoxicity.

In this regard, Pd-NP structural diversity and plasticity on supporting matrix [34,39] size, also in a very narrow distribution [51], chemical composition as well as surface functionalization [32,35], may all represent key NP properties potentially affecting their toxicological behavior. This may finally suggest the possibility to modify nanomaterial bioactivity changing NP physico-chemical characterization, therefore supporting the “safe by design” concept. This argues for nanomaterials that, while maintaining most of their innovative and revolutionary physico-chemical properties, are at the same time characterized by absent or lower toxicity. From an occupational health perspective, this may lead to a safer production and application of such materials, possibly preventing the development of adverse health effects.

Experiments carried out on animal models are extremely important to primarily define the toxico-kinetic and dynamic- behavior of NPs, as well as to identify possible biological indicators of exposure and early effect. Several organ systems have been reported to be affected by NP exposure, from the immune to the endocrine, as well as the renal systems [52,53,54,55,62]. However, it remains to be elucidated whether early alterations detected in sub-acute, sub-chronic conditions of exposure, i.e., cytokine changes, alteration in hormonal serum concentrations, as well as increase in urinary protein content, may persist in the long term, and their possible role in disease development and manifestation. Future research, in this perspective, should be focused on the health impact of low-dose, long term conditions of exposure, as those potentially experienced in real workplace settings.

Interestingly, concerning the immuno-modulatory effects of Pd-NPs on cytokine production and/or release, preliminary investigation on this topic in vivo [52,53] failed to show a significant preferential development of a specific cytokine secretion profile that may be responsible for an imbalance toward a cellular or humoral immune response. This may reflect the complexity of the Pd-NP interaction with biological systems in vivo which may be affected by modes of exposure, penetration of physiological barriers, solubility in biological media as well as the bio-molecular corona formation, which may all change the impact of the Pd-NP insult.

From an occupational health perspective, the possibility to extrapolate from animal investigations possible biomarkers of exposure and effects seems absolutely important. Considering the still existing practical difficulties in environmental monitoring of workplace NP exposure, due to the lack of standardized procedures and dosimetric parameters to be measured, biological monitoring may provide complementary information concerning exposure occurring by all routes, considering the inter-individual variabilities in the toxicokinetic of chemicals [63]. In this regard, the Pd elemental content in biological matrices should be investigated and validated as a possible indicator of internal dose in real occupational exposure settings. On the other side, alterations in nephrotoxic, immunological and hormonal parameters should be verified and confirmed as possible biomarkers of early effects in in field studies. The suitable definition of Pd-NP target organs, may be important to understand possible conditions of susceptibility due to pre-existing alterations/pathologies in such organs, which may deserve particular attention from an occupational health point of view [64].

## 7. Conclusions

This review represents the first attempt to achieve a more comprehensive knowledge of the toxicological profile of Pd-NPs. Although the potential for these nanomaterials to affect the ecosystem function, to exert cytotoxic and pro-inflammatory effects in vitro as well as to induce early alterations in different target organs in in vivo models has been reported, further investigations appear absolutely necessary to confirm such preliminary findings.

These should take advantage of a more comprehensive characterization of the physico-chemical properties of Pd-NPs in order to get a deeper understanding of the complex interplay between their intrinsic features and the detected health effects. Additionally, field studies strongly encourage defining the environmental levels of exposure to Pd-NPs in real workplace settings. Environmental assessment should be accompanied by biological monitoring investigations focused on verifying and validating experimental biomarkers of exposure and early effects in a real exposure contexts. Overall, this seems important to support the definition of adequate strategies for the evaluation of the risks derived from Pd-NP exposure, and, in a precautionary manner, to guide the adoption/implementation of comprehensive preventive and protective strategies to manage such emerging risks and protect the health and safety of workers.

## Figures and Tables

**Table 1:** Studies investigating the effects of Pd-NPs on in vitro models.

Palladium-NP Characterization	Investigated Models	Experimental Design	Endpoints	Results	References
**Plants**					
Size: 1–12 nm	Barley (*Hordeum vulgare*) plants	Seeds were cultivated in a floating hydroponic system containing a nutrient solution (0–50 μmol Pd/L) for 1 week	Leaf length	Leaf length significantly decreased according to the increased exposure concentrations (105–115 mm after 50 μmol Pd vs. 120–125 mm of unexposed plants).	Battke et al. [28]
Pd-NPs entrapped in an aluminum hydroxide matrix	Lettuce seeds	Seeds were planted immediately (day 0) or 15 day after adding NPs to the soil (0.013 and 0.066% *w/w*)	Seed germination and growth	Shoot/root ratio: no significant influence. on the difference in the ratio when the seeds were planted on day 0. palladium-NPs at low concentration significantly increased the ratio (2.5 cm) compared to controls (1.41 cm) in soils incubated with NPs for 15 d.	Shah and Bazelerova, [26]
Size: 5–10 nm	Kiwifruit pollen from plants of the male genotype of *Actinidia deliciosa var. deliciosa*	Pollen was exposed to 0–7 mg/L NPs for up to 90 min	Pollen performance and lethality	Tube emergence and growth: significant inhibition began at 0.1 mg/L, complete growth cessation at 0.4 mg/L. Lethality: as concentration increased, viability exponentially decreased (LC50 at 90 min: 1.0 ± 0.3 mg/L).	Speranza et al. [27]
Halloysite supported Pd-NPs. Tube diameter: 50 nm; Inner lumen diameter: 15 nm; Surface area: 65 m^2^/g	High and low vigor *R. sativus*	Radish seeds were exposed to 10 mL of nanomaterials at concentrations ranging 0–1500 mg/L for up to 72 h	Germination and seedling development	Exposure to Halloysite -PdNPs had no significant influence on germination, seedling development, xylem differentiation, or mitotic index in both lots.	Bellani et al. [39]
**Animal cells**					
Chemical composition: Pd/magnetite; Size: 20–30 nm; Purity: ≥98% Surface area: 46.4 ± 0.2 m^2^/g	RTgill-W1	Cells were exposed to 5–25 mg/L NPs for 1 h and 3 days	Cell viability	Metabolic activity and membrane integrity showed a significant decrease after 1 h exposure due to cellular stress. Cell viability was full restored after 3 days.	Hildebrand et al. [40]
Size: 10 ± 6 nm	Rat-1	Cells were exposed to 1 and 2 μg/mL NPs for 2–120 h	Cell viability; Oxidative stress reaction; cell cycle distribution; DNA damage	Cell viability was significantly reduced by both concentrations at 120 h. Cell cycle distribution: time-dependent increase in G0/G1 phase (from 45% in controls to 70% following 2 μg/mL for 120 h). Decrease of cell percentage in the S phase (from 30% in controls to 15% following 2 μg/mL for 120 h). DNA damage: a significant effect was evident only following treatment with 2 μg/mL for 120 h. ROS production: significant increase compared to controls only after 2 μg/mL NPs for 2 and 4 h.	Iavicoli et al. [30]
**Human cells**					
Size: 5–10 nm	PBMCs from 8 non-atopic healthy donors	Cells were overnight incubated with NPs (10^−5^, 10^−6^ M), with or without 10 μg/mL LPS stimulation	Cell viability; Cytokine release	Cell viability: not affected by all conditions of exposure. Cytokine release (no LPS): 10^−5^ M Pd-NPs significantly inhibited TNF-α and IL-17. Cytokine release (with LPS): 10^−5^ M Pd-NPs significantly inhibited TNF-α and IL-17 and increased INF-γ. Pd-NPs (10^−6^ M) significantly inhibited IL-17.	Boscolo et al. [37]
Size: 5–10 nm	PBMCs from 12 non-atopic and 8 atopic donors	Cells were overnight incubated with NPs (10^−5^ M), with or without 10 μg/mL LPS stimulation	Cell viability; Cytokine release	Cell viability: not affected by all conditions of exposure. Cytokine release (no LPS): no significant effects on cytokine release. Cytokine release (with LPS): NPs significantly reduced TNF-α and increased INF-γ levels in cells from non-atopic subjects. NPs reduced TNF-α in cells from atopic subjects.	Reale et al. [38]
Chemical composition: Pd/magnetite; Size: 20–30 nm; Purity: ≥98% Surface area: 46.4 ± 0.2 m^2^/g	Caco-2, Hacat	Cells were exposed to 5–25 mg/L NPs for 1 h and 3 days	Cell viability; Oxidative stress reaction	Hacat cells: viability was reduced to app. 91 ± 3% for all tested concentrations. After 3 days cell viability recovered up to 94% except with highest dose. Caco-2 cells showed similar tendency. No significant ROS production was demonstrated in comparison to controls.	Hildebrand et al. [40]
Size: 10.4 ± 2.7 nm; Shape: spherical	PBECs, A549	Cells were exposed to 0–25 μg/mL NPs for 24 h	Cell viability; Oxidative stress reaction; inflammatory response	Cell viability: no alterations up to 10 μg/mL, decreased by higher concentrations. PBEC apoptosis: dose-dependently induced. ROS: no significant induction. IL-8: significantly increased at 0.01 and 10 μg/mL, and decreased at 0.1 μg/mL in A549 cells; significantly decreased at 1 μg/mL for PBECs compared to controls. PGE_2_: significantly decreased at 0.1 and 10 μg/mL in A549 cells; significantly decreased at all the concentrations tested for PBECs compared to controls.	Wilkinson et al. [29]
Zerovalent NPs; Size: 2–8 nm	PBMCs from healthy donors	Cells were exposed to 0–80 μg/mL NPs for 4–72 h following PHA stimulation (48 h)	Cell viability; Oxidative stress reaction; Cell cycle distribution	No alterations in cell viability (4 h); Significant reduction at 80 (24 h), 20 (48 h) and 10 μg/mL (72 h). A moderate (n.s.) intracellular ROS increase was induced by NP exposure. Cell cycle distribution: PHA-stimulated PBMCs (G0/G1: 43.1 ± 1.9%; S: 48.8 ± 2.3%; G2/M: 8.1 ± 1.2%); 10 μg/mL NPs (G0/G1: 59.8 ± 1.8%; S: 35.4 ± 1.5%; G2/M: 4.8 ± 1.2%).	Petrarca et al. [31]
Size: 3.8 ± 0.4–26.6 ± 2.2 nm; Agglomerate size: 50–150 nm	THP-1	Cells were exposed to 0–450 μg/mL NPs	Oxidative stress reaction	A linear dependence of ROS production was evident up to 100 μg/mL. Above this level ROS levels were leveled off.	Neubauer et al. [41]
Pd-NPs obtained through leaf extracts of *Evolvulus alsinoides*. Shape: spherical; Size: 27 ± 2 nm	A2780	Cells were exposed to 0–10 μg/mL NPs for 24 h	Cell viability; Oxidative stress reaction	Cell viability: doses greater than 6 μg/mL NPs induced a significant reduction compared to controls. ROS production: dose-dependent, significant increase in treated cells compared to controls. Apoptosis: induced by NP treatment compared to controls.	Gurunathan et al. [4]
Pd nanosheets; positively charged CA-Pd nanosheets; Negatively charged 3-MPA-Pd-nanosheets	HepG2	Cells were incubated with nanosheets at 0–100 ppm for 24 h	Cell viability	Pd nano-sheets: 93.2 ± 6.8% at 50 ppm; 74.7 ± 8.5% at 100 ppm. CA-Pd nano-sheets: 42.4 ± 11.9% at 50 ppm; 17.93 ± 3.74% at 100 ppm. MPA-Pd nano-sheets: 83.7 ± 8.54% at 50 ppm; 68.4 ± 3.9% at 100 ppm.	Pan et al. [35]
Pd-NPs synthesized through leaf extract of *Eclipta prostrate*. Size: 18–64 nm (average size 27 ± 1.3 nm); Aggregate sizes: 63 ± 1.4 nm	HepG2	Cells were incubated with nanosheets at 0–500 μg/mL	Cell viability	Cytotoxicity was 8.5, 24, 48, 65, and 76.5% of cells treated with 1, 10, 100, 250, 500 μg/mL.	Rajakumar et al. [33]
Pd-nanocubes Size: ~10 nm	HeLa	Cells were exposed to 425 μg/mL Pd-nanocubes for 24 h	Cell viability	Percent of non-viable cells (necrotic or late apoptotic): 6.6% with serum, 14.7% without serum. Percent of apoptotic cells: ~7% with serum, 23% without serum (DNA fragmentation test).	Dahal et al. [34]
Pd-NPs and bimetallic Pt-Pd-NPs obtained through the extract of *Dioscorea bulbifera*. Pd-NP shape: spherical and blunt ended cubes; Pt-Pd-NP shape: irregular; Size: 10–25 nm	HeLa	Cells were exposed to 10 μg/mL NPs for 48 h	Cell viability	Reduced by 33.15% by Pd-NPs and by 74.25% by Pt-Pd-NPs. Immunofluorescence analysis indicated apoptosis of the treated cells (higher in geoups treated with bimetallic NPs than with Pd-NPs). Anti-scavenging activity: greater for bimetallic NPs than individual Pd-NPs.	Ghosh et al. [32]
Size: 10 ± 6 nm	A549	Cells were exposed to 1 and 2 μg/mL NPs for 4–120 h	Cell viability; Oxidative stress reaction; cell cycle distribution; DNA damage	Cell viability was significantly reduced by 2 μg/mL NPs at 96 and 120 h. Cell cycle distribution: time-dependent increase in G0/G1 phase (40% in controls to 80% following 2 μg/mL for 120 h). Decrease of cell percentage in the S phase (from 30% in controls to 15% following 2 μg/mL for 120 h). DNA damage: a significant effect was evident after 2 μg/mL at all time points. ROS production: significant increase compared to controls only for 2 μg/mL NPs for 4 h.	Iavicoli et al. [30]
Shape: spherical; Size: 6–10 nm Hydrodynamic diameter: 138 ± 3 and 151 ± 6 nm in ethanol and KSFM medium, respectively	Bronchial mucosa 3D model: PBECs and MRC-5	Bronchial cells [stimulated or not with IL-13 (1 ng/mL)] were exposed to aerosolized NPs at 250, 400 and 650 ng/cm^2^ for 24 h	Cell viability; Release of inflammatory mediators	No alterations in cell viability. IL-8 release: significantly increased in the basal medium following the medium dose exposure in the normal model and the highest concentration in IL-13 stimulated model, compared to controls.	Ji et al. [42]
Mean size (±SD): 14.70 ± 2.30 nm	A375	Cells were exposed to 0–40 μg/mL NPs for 24 and 48 h	Cell viability; Oxidative stress reaction; Cell cycle	Cell viability: dose- and time-dependent significant reduction (62.3% and 75.94% decrease at 24 and 48 h, compared to controls). ROS production: significantly increased by all concentrations of Pd-NPs. A significant reduction of GSH, 15.76% and 49.73%, compared to controls was induced by 40 μg/mL at 24 and 48 h, respectively. Cell cycle (24 h): cells in sub-G1 stage increased from 0.78% (untreated group) to 5.59% and 15.28%, at 20 μg/mL and 40 μg/mL concentrations, respectively.	Alarifi et al. [36]
Size: 1–10 nm; Hydrodynamic diameter in water: 446.9 ± 14.1 nm	Human eosinophilic cell line AML14.3D10 and freshly isolated eosinophils	Cells were incubated with 0–150 μg/mL NPs for up to 24 h	Cell viability; Oxidative stress reaction	Cell viability: no significant alterations in necrotic and apoptotic cells. ROS production: no significant alterations. Cytoskeleton rearrangement: actin was diffusely stained in the cytoplasm after NP treatment. Adhesion to endothelial cells: increased by a factor of 1.6 ± 0.2 (3D10 cell line treated with NPs); 1.7 ± 0.4 (freshly eosinophils treated with NPs) which are close to 1.8 ± 0.2 of positive controls.	Chhay et al. [43]

3D model, three dimensional model; A2780, human ovarian cancer cells; A375, human skin malignant melanoma cells; A549, transformed alveolar basal human epithelial cells; CA, cysteamine; Caco-2 cells, human colon adenocarcinoma cells; HaCat, human keratinocyte cells; HeLa, human epithelial cervical cancer cells; HepG2, human liver cancer cell line G2; LC50, lethal concentration 50; LPS, lypoplysaccharide; MPA, mercaptopropionic acid; n.s., not significant; MRC-5, human fetal lung fibroblast cells; NPs, nanoparticles; Pd, palladium; PBECs, primary bronchial epithelial cells; PGE_2_, prostaglandin E2; PHA, phytohemagglutinin-L; PBMCs, peripheral blood mononuclear cells; Rat-1, rat embryo fibroblasts; ROS, reactive oxygen species; SD, standard deviation; THP-1, human monocyte leukaemia cells.

**Table 2:** Studies investigating the effects of Pd-NPs on in vivo models.

Palladium-NP Characterization	Investigated Models	Experimental Designs	Endpoints	Results	References
**Bacterial models**					
Pd-NPs entrapped in an aluminum hydroxide matrix	Microcosm soil	NPs were added to the soil at a final concentration of 0.013% or 0.066% (*w/w*)	Bacterial growth	No significant effects on the number of colony forming units or on the total soil community metabolic fingerprint.	Shah and Belozerova, [27]
Bimetallic Pd-iron NPs	*Sphingomonas* sp. PH-07	Bacterial strain was exposed to 0–0.5 g/L	Bacterial growth	No differences compared to controls were evident up to 0.1 g/L, whereas bacterial growth was significantly inhibited with greater NP concentrations.	Murugesan et al. [56]
Size: 2.0 ± 0.1; 2.5 ± 0.2; 3.1 ± 0.2 nm.	Gram negative *E. Coli*, Gram positive *S. aureus* bacterial strains	Bacteria were exposed to 2.5 × 10^−4^, 10^−5^, 10^−6^, 10^−7^, 10^−8^, and 10^−9^ M for each size of NPs for 24 h	Bacterial growth	A significant decrease in the amount of colonies/mL over the 24 h exposure time, at the 10^−4^–10^−6^ M concentrations was evident for both microorganisms.	Adams et al. [51]
Pt-Pd polyaniline nanocomposites	Gram positive (*Streptococcus* sp. and *Staphylococcus* sp.) and Gram-negative bacteria (*E. coli* and *Klebsiella* sp.)	Bacteria were incubated with 0–150 μg/mL pristine anyline, Pt-Pd bimetal NPs and Pt-Pd polyaniline nanocomposites for 24 h	Bacterial growth	The maximum zone of inhibition (mm in diameter) was observed for polyaniline/Pt-Pd nanocomposite against *Staphylococcus* sp. (28 ± 0.70 mm) followed by *Klebsiella* sp. (25 ± 0.85 mm), *E. coli* (22 ± 0.36 mm) and *Streptococcus* sp. (21 ± 0.30 mm). MIC: 25 and 75 μg/mL for anyline and Pt-Pd nanocomposites. Pt-Pd colloids did not show the zone of inhibition against investigated pathogens.	Boomi et al. [57]
Pd-NPs obtained throuh methanolic extraction from *M. oleifera* peel. Shape: spherical; Size: 27 ± 2 nm	Gram negative *E. coli*, Gram positive *S. aureus* bacterial strains	-	Bacterial growth	Good antibacterial activity was reported for both Gram positive and Gram negative bacterial strains.	Surendra et al. [58]
Pd-NPs obtained through bio-precipitation in bacterial cultures	Marine Gram negative bacterium *V. fischeri*; PCB-dechlorinating marine microbial community cultures from a sediment collected in the Venice lagoon, Italy	*V. fischeri* were exposed to 0–9 μg/mL for 5–30 min. Marine cultures were exposed to 5 and 50 mg/kgdw for upt to 18 weeks	Bacterial growth of *V. fischeri*; Respiratory metabolisms and structure of the marine microbial community	Acute toxicity: no significant effects. Respiratory metabolisms: Pd-NPs did not impact the dechlorination activities; while dose-dependently inhibited the sulfate reduction and methanogenesis. Pd-NPs increased the richness of the microbial community.	Nuzzo et al. [59]
Pd-NPs obtained through leaf extract of *Garciniapedunculata*; Shape: spherical and non-spherical; Size: ~2 ± 4 nm; Hydrodynamic diameter: 322 nm	MDR clinical isolate *Cronobacter sakazakii* strain AMD04	Bacterial cultures (1 mL) were incubated with 0–0.65 mM NPs up to 24 h	Bacterial and biofilm growth	Bactericidal activity: a 31.67 ± 1.53 mm diameter zone of inhibition was evident around the Pd-NP well. MIC: 0.06 mM; MBC: 0.12 mM. Biofilm activity: maximum inhibition at 0.26 ± 0.02 mM. Comparable biomass formation decrease at 0.39 and 0.52 mM.	Hazarika et al. [60]
**Animal models**					
Size: 10 ± 6 nm	Female Wistar rats (n. 4 per group)	Animals were exposed to a single intravenous injection of Pd-NPs at 0–12 µg/kg dose. Serum cytokine concentrations were assessed at the 14th day post-exposure	Effects on the immune system	The highest dose of Pd-NPs (12 μg/kg) induced a significant increase of IL-1α, IL-4, IL-6, IL-10, IL-12, GM-CSF and INF-γ compared to controls. The 0.12 μg/kg dose induced a significant increase of IL-2, IL-4, IL-10 serum concentrations.	Iavicoli et al. [52]
	Female Wistar rats (n. 5 per group)	Animals were exposed to repeated (on day 1, 30 and 60) intravenous injections of Pd-NPs at 0–12 µg/kg dose. Serum cytokine concentrations were assessed at the 90th day post-exposure	Effects on the immune system	The highest dose of Pd-NPs (12 μg/kg) induced a significant reduction of IL-1α, IL-4, IL-6, IL-10, IL-12, and GM-CSF compared to controls. The 1.2 μg/kg dose induced also a significant reduction of IL-1α. Interleukin-10 was significantly reduced at all the investigated concentrations.	Iavicoli et al. [53]
	Female Wistar rats (n. 5 per group)	Animals were exposed to a single intravenous injection of Pd-NPs at 0–12 µg/kg dose. Urinary protein concentrations were analyzed at the 14th day post exposure	Effects on the renal system	RBP: significantly increased in the 12 µg/kg group (0.23 ± 0.07 mg/mL) compared to controls (0.11 ± 0.03 mg/mL). Beta2-microglobulin: significantly increased in the 12 µg/kg group (0.97 ± 0.23 mg/mL) compared to controls (0.40 ± 0.07 mg/mL).	Fontana et al. [54]
	Female Wistar rats (n. 4 per group)	Animals were exposed to a single intravenous injection of Pd-NPs at 0–12 µg/kg dose. E2, FSH, LH, P and T levels were determined in the serum samples collected 14 days after exposure	Effects on the endocrine system	Dose 12 μg/kg: a significant reduction of E2 (0.012 ± 0.002 ng/mL) and T (0.47 ± 0.04 ng/mL) concentrations and an increase of LH levels (23.09 ± 1.07 ng/mL) compared to controls (E2: 0.032 ± 0.006; T: 0.80 ± 0.76; LH: 16.48 ± 1.31 ng/mL). Dose 1.2 μg/kg: significant reduction for E2 (0.012 ± 0.005 ng/mL) and increase for LH (20.11 ± 1.14 ng/mL) compared to controls.	Leso et al. [55]

Caco-2 cells, human colon adenocarcinoma cells; E2, estradiol; FSH, follicle-stimulating hormone; GM-CSF, granulocyte-macrophage colony stimulating factor; HaCat cells, human keratinocyte cells; IL, interleukin; INF-γ, interferon-γ; LH, luteinizing hormone; MBC, minimum bactericidal concentration; MDR, multidrug resistant; MIC, minimum inhibitory concentration; NPs, nanoparticles; P, progesterone; RBP, retinol binding protein; T, testosterone.

## Abbreviations

3D model	Three dimensional model
A2780	Human ovarian cancer cells
A375	Human skin malignant melanoma cells
A549	Immortalized alveolar basal human epithelial cells
CA	Cysteamine
Caco-2	Human colon adenocarcinoma cells
E2	Estradiol
FSH	Follicle-stimulating hormone
GM-CSF	Granulocyte-macrophage colony stimulating factor
Hacat	Human keratinocyte cells
HeLa	Human epithelial cervical cancer cells
HepG2	Human liver cancer cells G2
IL	Interleukin
INF-γ	Interferon-γ
LC50LH	Lethal concentration 50Luteinizing hormone
LPS	Lypopolysaccharide
MBC	Mimum bactericidal concentration
MDR	Multidrug resistant
MIC	Minimum inhibitory concentration
MPA	Mercaptopropionic acid
n.s.	Not significant
MRC-5	Human fetal lung fibroblast cells
NPs	Nanoparticles
P	Progesterone
Pd	Palladium
PBECs	Primary bronchial epithelial cells
PGE_2_	Prostaglandin E2
PHA	Phytohemagglutinin-L
PBMCs	Peripheral blood mononuclear cells
Pt	Platinum
Rat-1	Rat embryo fibroblasts
RBPROS	Retinol binding proteinReactive oxygen species
SD	Standard deviation
T	Testosterone
THP-1	Human monocyte leukaemia cells
